# When can maximal efficacy occur with repeat botulinum toxin injection in upper limb spastic paresis?

**DOI:** 10.1093/braincomms/fcaa201

**Published:** 2020-11-18

**Authors:** Jean-Michel Gracies, Robert Jech, Peter Valkovic, Philippe Marque, Michele Vecchio, Zoltan Denes, Claire Vilain, Bruno Delafont, Philippe Picaut

**Affiliations:** 1 UR 7377 BIOTN, Université Paris-Est Créteil, Service de Rééducation Neurolocomotrice, Hôpitaux Universitaires Henri Mondor, Créteil, France; 2 Department of Neurology and Center of Clinical Neuroscience, Charles University, First Faculty of Medicine and General University Hospital, Prague, Czech Republic; 3 2nd Department of Neurology, Comenius University Faculty of Medicine and University Hospital Bratislava, Bratislava, Slovak Republic; 4 Service de médecine physique et réadaptation, Hôpital Rangueil, Toulouse, France; 5 Department of Biomedical and Biotechnological Sciences, University of Catania, Catania, Italy; 6 Physical Medicine and Rehabilitation Unit, A.O.U. Policlinico Vittorio Emanuele, Catania, Italy; 7 Brain Injury Rehabilitation Unit, National Institute for Medical Rehabilitation, Budapest, Hungary; 8 Ipsen Pharma, Les Ulis, France; 9 Delafont Statistics, Alençon, France

**Keywords:** abobotulinumtoxinA, repeat injection, maximal effect, non‐linear random coefficient, upper limb spastic paresis

## Abstract

Repeated injection cycles with abobotulinumtoxinA, a botulinum toxin type A, are recommended in current clinical guidelines as a treatment option for adults with upper limb spastic paresis. However, the magnitude of the maximal therapeutic effect of repeated abobotulinumtoxinA treatment across different efficacy parameters and the number of injection cycles required to reach maximal effect remain to be elucidated. Here, we present a *post hoc* exploratory analysis of a randomized, double-blind, placebo-controlled trial (12–24 weeks; NCT01313299) and open-label extension study (up to 12 months; NCT0131331), in patients aged 18–80 years with hemiparesis for ≥6 months after stroke/traumatic brain injury. Two inferential methods were used to assess the changes in efficacy parameters after repeat abobotulinumtoxinA treatment cycles: Mixed Model Repeated Measures analysis and Non-Linear Random Coefficients analysis. Using the latter model, the expected maximal effect size (not placebo-controlled) and the number of treatment cycles to reach 90% of this maximal effect were estimated. Treatment responses in terms of passive and perceived parameters (i.e. modified Ashworth scale in primary target muscle group, disability assessment scale for principal target for treatment or limb position, and angle of catch at fast speed) were estimated to reach near-maximal effect in two to three cycles. Near-maximal treatment effect for active parameters (i.e. active range of motion against the resistance of extrinsic finger flexors and active function, assessed by the Modified Frenchay Scale) was estimated to be reached one to two cycles later. In contrast to most parameters, active function showed greater improvements at Week 12 (estimated maximal change from baseline-modified Frenchay Scale overall score: +0.8 (95% confidence interval, 0.6; 1.0) than at Week 4 (+0.6 [95% confidence interval, 0.4; 0.8]). Overall, the analyses suggest that repeated treatment cycles with abobotulinumtoxinA in patients chronically affected with upper limb spastic paresis allow them to relearn how to use the affected arm with now looser antagonists. Future studies should assess active parameters as primary outcome measures over repeated treatment cycles, and assess efficacy at the 12-week time-point of each cycle, as the benefits of abobotulinumtoxinA may be underestimated in the studies of insufficient duration.

**Abbreviated summary**

In this *post hoc* analysis of repeated abobotulinumtoxinA injection cycles in upper limb spastic paresis, Gracies *et al.* used statistical modelling to elucidate the maximal therapeutic effect of abobotulinumtoxinA. Notably, the number of injections required to reach this maximal effect was higher for active (e.g. active function) compared with passive (e.g. tone) parameters.

## Graphical Abstract

**Graphical Abstract:**
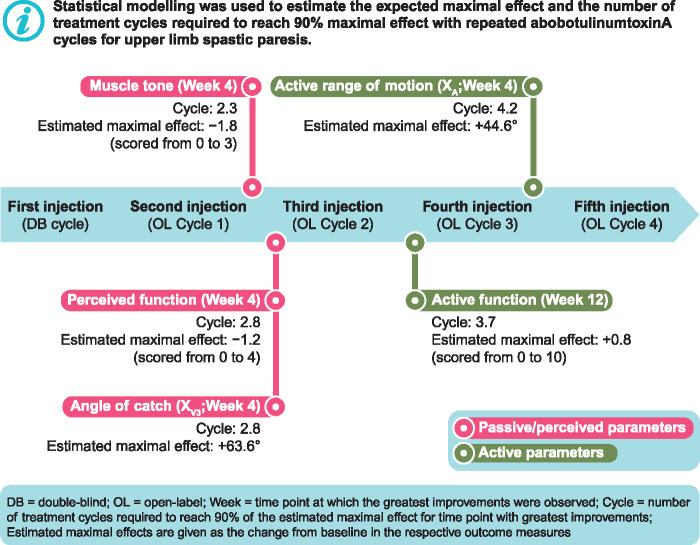

## Introduction

Placebo-controlled clinical trials in adults with chronic spastic paresis of the upper limb as a result of multiple aetiologies (e.g. stroke or traumatic brain injury) have demonstrated that abobotulinumtoxinA (Dysport^®^, Ipsen Biopharm, Wrexham, UK) treatment effectively reduces muscle tone and spasticity, and enhances perceived function and active range of motion (Dashtipour *et al.*, 2015). Furthermore, these benefits have been shown to be maintained and improvements in active function have been observed with repeated treatment cycles (Gracies *et al.*, 2018). Current clinical guidelines recommend abobotulinumtoxinA as a treatment option for spastic paresis of the upper limb in adult patients (Simpson *et al.*, 2016; Royal College of Physicians, 2018). However, some questions remain unanswered: are there indefinitely incremental improvements with repeated abobotulinumtoxinA administrations, or do treatment effects plateau after a certain number of injections? If a maximal effect is expected with repeated injections, what is its magnitude, and can the same pattern of effect be observed for all efficacy parameters, or does it vary depending on the parameter considered? Resolving these questions will help physicians plan rehabilitation and injection visits, manage patient expectations and ensure realistic timeframes for patients’ goals.

A recent preliminary statistical investigation by Delafont *et al.* (2018) assessed treatment responses to repeated abobotulinumtoxinA injections in patients with upper limb spastic paresis, using data from a single-cycle, randomized, double-blind, placebo-controlled trial (NCT01313299) (Gracies *et al.*, 2015) and an open-label extension study of up to 12 months (NCT0131331) (Gracies *et al.*, 2018). Two statistical models (non‐linear random coefficient [NLRC] and mixed-model repeated measures [MMRM]) were used to estimate responses to treatment at Week 4 across treatment cycles. The NLRC model, which assumed a negative exponential shape for changes in efficacy parameters over time, was used to additionally estimate the expected maximal treatment effect and number of treatment cycles required to achieve 90% of the maximal treatment effect (deemed a clinically relevant threshold). Treatment effects were assessed in terms of two efficacy parameters: the technical assessment ‘active range of motion (X_A_)’ against the resistance of extrinsic finger flexors, and the assessment of active function ‘overall Modified Frenchay Scale (MFS) score’ (Gracies *et al.*, 2010). This previous investigation verified that the NLRC model could be used to estimate maximal efficacy and the treatment duration required to reach it as long as analysis results did not indicate a violation of its assumptions (Delafont *et al.*, 2018).

To further investigate responses to repeated abobotulinumtoxinA treatment, this exploratory analysis used the same statistical modelling to again estimate the expected maximal therapeutic effect of repeat abobotulinumtoxinA injections and number of treatment cycles required to reach 90% of maximal treatment effect, but for a range of additional efficacy parameters, at two time points post-injection.

## Materials and methods

### Study design

A retrospective, *post hoc* exploratory analysis of a subset of data from two clinical studies of abobotulinumtoxinA was performed: a phase 3, double-blind, placebo-controlled trial of a single-treatment cycle (12–24 week duration; NCT01313299) (Gracies *et al.*, 2015), followed by an open-label extension study (multiple treatment cycles, up to 12 months of treatment, with a minimum of 12 weeks between injections) (NCT01313312) (Gracies *et al.*, 2018).

Full methodology and the primary results from both trials have been published previously (Gracies *et al.*, 2015, 2018). In brief, eligible patients were men and women aged 18–80 years, with hemiparesis for ≥6 months after a stroke or traumatic brain injury (Gracies *et al.*, 2015). In the double-blind trial, patients were randomized (1:1:1) to either abobotulinumtoxinA 500 Units (U), abobotulinumtoxinA 1000 U or placebo (Gracies *et al.*, 2015). Patients who completed the double-blind trial without ongoing adverse events or major protocol deviations were eligible for the open-label extension study (Gracies *et al.*, 2018), which also enrolled newly recruited patients. The present analysis was performed on a subset of the extension study intention-to-treat population (intention-to-treat: all patients who received ≥1 abobotulinumtoxinA injection in the open-label study), who received abobotulinumtoxinA treatment (regardless of dose) during the initial double-blind trial. Newly recruited patients to the extension study, and patients receiving placebo in the double-blind trial, were excluded from this analysis.

In the extension study, rollover patients from the double-blind trial could receive up to four additional treatment cycles of 500, 1000 or 1500 U abobotulinumtoxinA (dosage at investigator’s discretion, dependent on patients’ need; for the 1500 U dose, it was stipulated 500 U must be injected into shoulder extensors, with a maximum 1000 U across finger, wrist and elbow flexors). Dose groups were combined for this analysis, as doses could vary within each patient across treatment cycles.

### Description and classification of efficacy parameters

All efficacy parameters were assessed in terms of their change from baseline (baseline = value at the start of the double-blind trial) at Week 4 or 12 of each treatment cycle. Efficacy parameters were classified as ‘technical’ or ‘functional’ assessments, and were also considered in terms of being ‘passive/perceived’ or ‘active’. For reference, ‘technical’ assessments belong to the wider World Health Organization ‘International Classification of Functioning, Disability and Health-body functions’ domain, whereas ‘functional’ assessments, including those for ‘perceived’ function, belong to the wider category of the ‘International Classification of Functioning, Disability and Health-activities and participation’ domain (World Health Organization, 2001).

The following technical assessments were investigated: (i) muscle tone (using the modified Ashworth scale [MAS] in the primary target muscle group [PTMG], i.e. most affected upper limb muscle group, selected from extrinsic finger, wrist or elbow flexors; 0 [no increase in muscle tone] to 4 [rigid in flexion or extension]); (ii) Tardieu scale for angle of arrest at slow speed (passive range of motion, *X*_V1_) and angle of catch at fast speed (*X*_V3_), both assessed against the resistance of extrinsic finger, wrist and elbow flexors; (iii) *X*_A_ against the resistance of extrinsic finger, wrist and elbow flexors (Gracies *et al.*, 2010).

The following functional assessments were investigated: (i) active function evaluated by the MFS overall score (mean score of 10 tasks rated on a 10-point visual analogue scale) (Gracies *et al.*, 2010); (ii) perceived function evaluated by the Disability Assessment Score (DAS; 0 [no disability] to 3 [severe disability]) for the principal target for treatment (PTT; either limb position, dressing, hygiene or pain) and the DAS for limb position (Brashear *et al.*, 2002). To assess differences in active function by patients’ age groups, MFS overall score was also investigated in patients above or equal to/below the median age of the population.

### Statistical analysis

#### Statistical models

Two inferential methods were used to estimate the effects of treatment over time: an NLRC analysis and an MMRM analysis. In brief, the NLRC analysis allows for estimation of the maximal treatment effect and time to 90% of maximal treatment effect; however, it is not expected to reliably model all efficacy parameters. Therefore, the MMRM was used as a reference method to gauge the accuracy of the NLRC analysis, as it is more flexible in terms of assumptions. Separate analyses were run for each efficacy parameter and each post-injection time point of interest (Weeks 4 and 12).

The NLRC model assumed that changes in efficacy parameters (to Week 4 or 12 over repeated treatment cycles) followed a negative exponential function of the number of treatment cycles. The asymptote and rate parameters of the negative exponential function were included as random coefficients and assumed to follow a bivariate normal distribution. The maximal effect size expected after a given number of treatment cycles was determined using the estimated asymptote. The number of treatment cycles required to reach 90% of the maximal effect was derived using the negative exponential rate parameter.

The MMRM is widely used for modelling longitudinal data, especially in the presence of missing dependent data (Prakash *et al.*, 2008). For each efficacy parameter, the change from baseline (to Week 4 or 12) was included as a dependent variable, patient was included as a random effect and treatment cycle as a fixed effect with a heterogeneous first-order autoregressive covariance structure. Unlike the NLRC, the MMRM model does not assume any specific shape of the response curve over time.

If the two models clearly diverged or if NLRC residuals indicated a clear bias, the MMRM analysis was chosen as the default method due to its more flexible assumptions, in which case the NLRC results were discarded. In the presence of smaller differences between models, both statistical and medical judgements were considered to prefer one method over the other. However, the MMRM does not allow for the determination of the maximal treatment effect or related parameters.

Full details of the two models, and a rationale for their use have been published previously (Delafont *et al.*, 2018). Both models were implemented using SAS statistical package v9.4.

#### Presentation of analyses

The change from baseline at Weeks 4 and 12 of each treatment cycle was estimated using least-squares means, with the corresponding 95% confidence interval (CI). To facilitate comparisons between model estimates over time, least-squares means from both models were plotted simultaneously. For the NLRC analysis, the asymptote and number of treatment cycles to reach 90% of the asymptote were estimated with 95% CIs. For convenience, the asymptote has been included in the least-squares means plots.

## Data availability

Where patients’ data can be anonymized, Ipsen will share all individual participant’s data that underlie the results reported in this article with qualified researchers who provide a valid research question. Study documents, such as the study protocol and clinical study report, are not always available. Proposals should be submitted to DataSharing@Ipsen.com and will be assessed by a scientific review board. Data are available beginning 6 months and ending 5 years after publication; after this time, only raw data may be available.

## Results

### Baseline characteristics

Overall, 152 patients received abobotulinumtoxinA treatment during the double-blind cycle and at least one cycle of abobotulinumtoxinA during the open-label phase. Baseline characteristics are available in the primary double-blind and open-label extension publications (Gracies *et al.*, 2015, 2018). In brief, for patients receiving abobotulinumtoxinA at double-blind study baseline, the mean (SD) age was 52.8 (13.2) years (median [range], 55.0; [18, 76] years), and 65.4% of patients were male. Baseline characteristics by age group (≤55 years and >55 years) are summarized in Supplementary Table 1. The group aged ≤55 years had a greater proportion of patients with spasticity due to traumatic brain injury (13.6%) compared with those >55 years (4.2%), and appeared to be more severely affected, with lower mean and median baseline scores for MFS and *X*_A_ against the resistance of extrinsic finger, wrist and elbow flexors, compared with the older age group.

### Efficacy parameters

In line with the preliminary investigation (Delafont *et al.*, 2018), the NLRC successfully estimated the maximal treatment response and number of cycles required for a near-maximal response for the majority of technical and functional efficacy parameters investigated. There were, however, some instances where the results of NLRC analysis were questionable due to a suspected violation of its modelling assumption.

#### Technical assessments

##### Modified Ashworth Scale in the primary target muscle group

**Figure 1:**
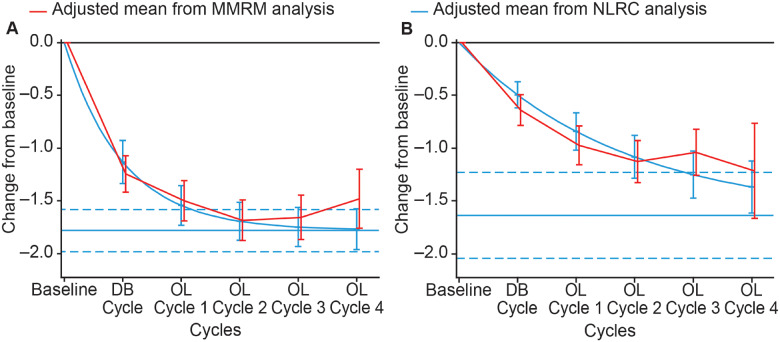
**Estimated mean change from baseline Modified Ashworth Scale score for the primary targeted muscle group.** (**A**) At Week 4 of treatment cycle. (**B**) At Week 12 of treatment cycle. Displayed intervals are 95% CIs. Horizontal blue lines correspond to the estimated asymptote from NLRC model, and its 95% CI. CI = confidence interval; DB = double blind; MMRM = mixed model repeated measures; NLRC = non‐linear random coefficient; OL = open label

At Week 4 of any treatment cycle, the estimated maximal change from baseline MAS score in the PTMG was −1.8 (95% CI, –2.0; –1.6) (Fig. 1A), with 90% of the asymptote reached after an estimated 2.3 (95% CI, 1.7; 3.3) cycles; at Week 12 the estimated maximal change from baseline was −1.6 (95% CI, –2.0; –1.2) (Fig. 1B), with 90% of the asymptote reached after an estimated 6.4 (95% CI, 4.5; 10.9) cycles. There was some divergence of the estimates from the two models at later cycles, however, in the absence of any clear violation of NLRC assumptions, both models were deemed appropriate for the assessments (Fig. 1A and B).

##### Passive range of motion (*X*_V1_) and angle of catch at fast speed (*X*_V3_) in extrinsic finger flexors

**Figure 2:**
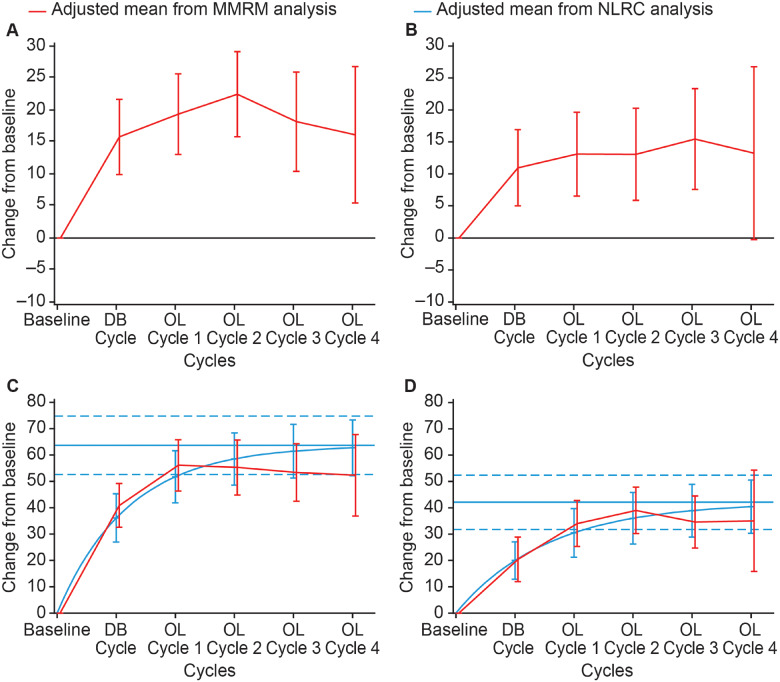
**Estimated mean change from baseline passive range of motion (X_V1_) and angle of catch at fast speed (X_V3_) (Tardieu scale) in the extrinsic finger flexors.** (**A**) *X*_V1_ at Week 4 of treatment cycle. (**B**) *X*_V1_ at Week 12 of treatment cycle. (**C**) *X*_V3_ at Week 4 of treatment cycle. (**D**) *X*_V3_ at Week 12 of treatment cycle. Displayed intervals are 95% CIs. Horizontal blue lines correspond to the estimated asymptote from NLRC model, and its 95% CI. CI = confidence interval; DB = double blind; MMRM = mixed model repeated measures; NLRC = non‐linear random coefficient; OL = open label

The NLRC model fit of change from baseline *X*_V1_ in extrinsic finger flexors at Weeks 4 and 12 of a treatment cycle was questionable and the maximal change from baseline could not be determined. Only the MMRM model was deemed appropriate for the estimation of this efficacy parameter (Fig. 2A and B).

The estimated maximal change from baseline *X*_V3_ (Tardieu scale) in extrinsic finger flexors at Week 4 of a treatment cycle was 63.6° (95% CI, 52.6; 74.7) (Fig. 2C), with 90% of the asymptote reached after an estimated 2.8 (95% CI, 2.0; 4.5) cycles. At Week 12, the estimated maximal change in *X*_V3_ for finger flexors was 42.1° (95% CI, 31.8; 52.4) (Fig. 2D), with 90% of the asymptote reached after an estimated 3.6 (95% CI, 2.6; 5.6) cycles. Small differences between the estimates of this parameter from the two models were observed at later cycles. Although the NLRC model produced higher estimated values than the MMRM, both models were considered appropriate methodological approaches for the estimation of the change from baseline *X*_V3_ at Weeks 4 and 12 of a treatment cycle.

##### Passive range of motion (*X*_V1_) and angle of catch at fast speed (*X*_V3_) in wrist flexors

For *X*_V1_ in wrist flexors, the estimated maximal change from baseline at Week 4 of a treatment cycle was 18.2° (95% CI, 12.7; 23.7), with 90% of the asymptote reached after an estimated 4.7 (95% CI, 3.3; 8.1) cycles. The estimated maximal change from baseline *X*_V1_ at Week 12 was 13.0° (95% CI, 8.1; 17.9), with 90% of the asymptote reached after an estimated 4.1 (95% CI, 2.8; 7.8) cycles. For *X*_V3_, the estimated maximal change from baseline at Week 4 was 47.9° (95% CI, 39.6; 56.1), with 90% of the asymptote reached after an estimated 3.3 (95% CI, 2.4; 5.1) cycles. At Week 12, the estimated maximal change from baseline in *X*_V3_ was 33.3° (95% CI, 24.7; 41.9), with the 90% asymptote threshold reached after 4.5 (95% CI, 3.1; 7.8) cycles.

Some differences between the model-estimated changes from baseline were apparent between the models, in particular *X*_V1_ at Week 4 after open-label Cycle 1, and *X*_V1_ and *X*_V3_ at Weeks 4 and 12 after open-label Cycle 4, however, during other cycles both models yielded similar estimated values.

##### Active range of motion (*X*_A_) against the resistance of extrinsic finger flexors

**Figure 3:**
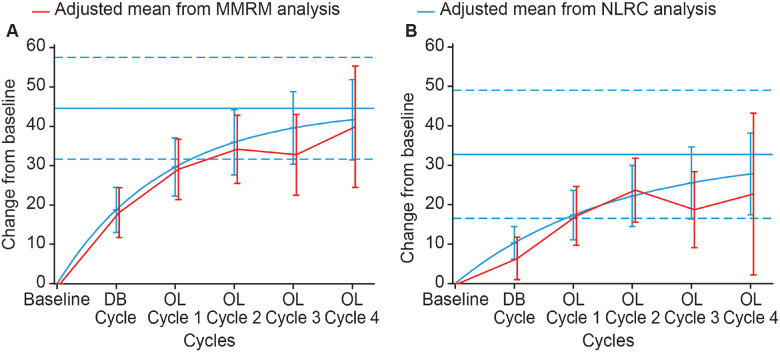
**Estimated mean change from baseline in the active range of motion (*X*_A_).** (**A**) In extrinsic finger flexors at Week 4 of treatment cycle. (**B**) In extrinsic finger flexors at Week 12 of treatment cycle. Displayed intervals are 95% CIs. Horizontal blue lines correspond to the estimated asymptote from NLRC model, and its 95% CI. CI = confidence interval; DB = double blind; MMRM = mixed model repeated measures; NLRC = non‐linear random coefficient; OL = open label

At Week 4 of a treatment cycle, the estimated maximal change from baseline *X*_A_ against extrinsic finger flexors was 44.6° (95% CI, 31.5; 57.6) (Fig. 3A), with 90% of the asymptote reached after an estimated 4.2 (95% CI, 2.8; 8.5) cycles; at Week 12 the estimated maximal change was 32.8° (95% CI, 16.5; 49.0) (Fig. 3B), with the 90% asymptote reached after 6.1 (95% CI, 3.7; 18.1) cycles. Some differences in the model-estimated change from baseline at Week 4 were apparent between the models for open-label Cycle 3 (Fig. 3A), and at Week 12 for open-label Cycles 3 and 4 (Fig. 3B). However, the 95% CI of the estimates from both models overlapped at every cycle, and both models were deemed appropriate for the estimation of changes in *X*_A_ against extrinsic finger flexors.

##### Active range of motion (*X*_A_) against the resistance of wrist flexors

The NLRC model for estimated change from baseline *X*_A_ against wrist flexors at Week 4 of a treatment cycle was questionable; therefore, the maximal change from baseline could not be determined and the MMRM was considered more appropriate for the assessment. At Week 12, the estimated maximal change from baseline *X*_A_ against wrist flexors was 27.8° (95% CI, 6.9; 48.8), with 90% of the asymptote reached after an estimated 12.5 (95% CI, 6.4; 231.0) cycles. Both models were deemed appropriate for the estimation of change from baseline *X*_A_ against wrist flexors at Week 12 of a treatment cycle.

##### Active and passive range of motion against the resistance of elbow flexors

The NLRC model for changes in *X*_A_ and *X*_V1_ in the elbow flexors at Weeks 4 and 12 was questionable. For these parameters, MMRM-estimates were used by default and the results are summarized in Table 1.

**Table 1:** Change from baseline in active range of motion (*X*_A_) and passive range of motion (*X*_V1_) against the resistance of elbow flexors

	Week 4		Week 12	
	*n*	MMRM estimates, mean (95% CI)	*n*	MMRM estimates, mean (95% CI)
*X*_A_, estimated change from baseline				
DB cycle	92	11.32 (6.79; 15.85)	91	2.84 (−3.81; 9.49)
OL Cycle 1	92	10.67 (5.89; 15.45)	85	5.21 (−0.27; 10.70)
OL Cycle 2	79	15.07 (10.85; 19.30)	81	8.62 (4.06; 13.17)
OL Cycle 3	52	13.91 (8.52; 19.30)	52	10.55 (4.63; 16.48)
OL Cycle 4	20	14.75 (7.39; 22.11)	19	10.66 (2.34; 18.97)
*X*_V1_, estimated change from baseline				
DB cycle	109	1.44 (0.31; 2.56)	107	0.76 (−0.95; 2.48)
OL Cycle 1	112	−0.51 (−2.84; 1.82)	108	0.36 (−1.93; 2.65)
OL Cycle 2	102	1.45 (−0.47; 3.36)	101	–1.34 (−2.94; 0.25)
OL Cycle 3	68	1.84 (−0.04; 3.73)	67	0.31 (−1.78; 2.39)
OL Cycle 4	24	0.11 (−2.34; 2.56)	21	–2.28 (−5.31; 0.74)

Abbreviations: CI = confidence interval; DB = double-blind; MMRM = mixed model repeated measures; OL = open-label.

#### Functional assessments

##### Modified Frenchay Scale overall score

**Figure 4:**
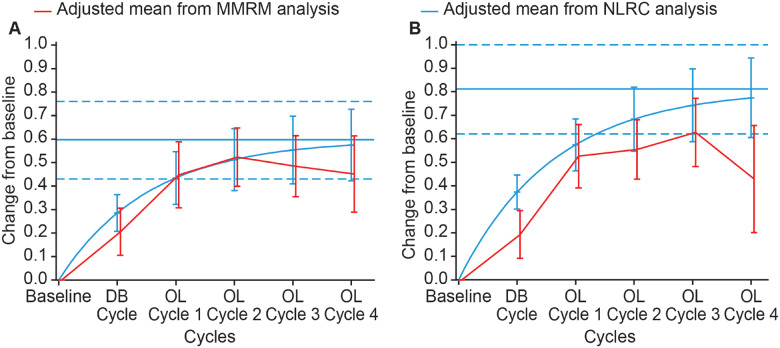
**Estimated mean change from baseline MFS overall score.** (**A**) At Week 4 of treatment cycle. (**B**) At Week 12 of treatment cycle. Displayed intervals are 95% CIs. Horizontal blue lines correspond to the estimated asymptote from NLRC model, and its 95% CI. CI = confidence interval; DB = double blind; MMRM = mixed model repeated measures; NLRC = non‐linear random coefficient; OL = open label

The estimated maximal change from baseline MFS overall score at Week 4 of any treatment cycle was 0.6 (95% CI, 0.4; 0.8) (Fig. 4A), with 90% of the asymptote reached after an estimated 3.5 (95% CI, 2.8; 4.9) cycles. The estimated maximal change from baseline at Week 12 was 0.8 (95% CI, 0.6; 1.0) (Fig. 4B), with 90% of the asymptote reached after an estimated 3.7 (95% CI, 3.0; 5.0) cycles. At Week 4, both model estimates provided similar assessments; however, some differences in the model estimates were observed at Week 12 with NLRC estimates consistently higher than MMRM estimates, despite these both models were deemed relevant and shown in in Fig. 4B.

##### Disability Assessment Score for PTT

**Figure 5:**
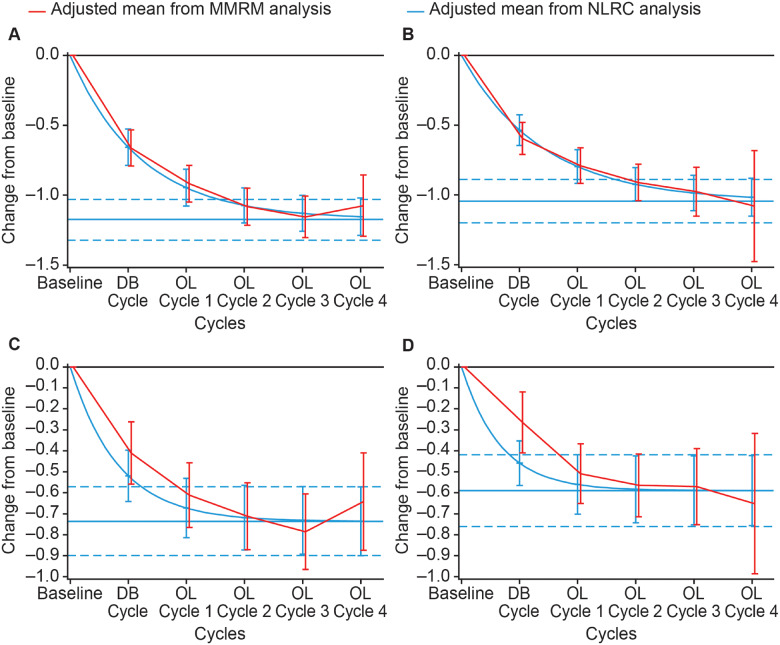
**Estimated mean change from baseline DAS score.** (**A**) For principal target of treatment at Week 4 of treatment cycle. (**B**) For principal target of treatment at Week 12 of treatment cycle. (**C**) For limb position at Week 4 of treatment cycle. (**D**) For limb position at Week 12 of treatment cycle. Displayed intervals are 95% CIs. Horizontal blue lines correspond to the estimated asymptote from NLRC model, and its 95% CI. CI = confidence interval; DB = double blind; MMRM = mixed model repeated measures; NLRC = non‐linear random coefficient; OL = open label

The estimated maximal change from baseline DAS for PTT at Week 4 of a treatment cycle was −1.2 (95% CI, −1.3; –1.0) (Fig. 5A), with 90% of the asymptote reached after an estimated 2.8 (95% CI, 2.1; 4.1) cycles. The estimated maximal change from baseline at Week 12 was −1.0 (95% CI, −1.2; –0.9) (Fig. 5B), with 90% of the asymptote reached after an estimated 3.2 (95% CI, 2.4; 4.9) cycles. Both models produced similar estimates for this parameter at Weeks 4 and 12 of the treatment cycles (Fig. 5A and B).

##### Disability Assessment Score for limb position

The estimated maximal change from baseline DAS score for limb position at Week 4 of a treatment cycle was −0.7 (95% CI, −0.9; –0.6) (Fig. 5C), with 90% of the asymptote (95% CI) reached after an estimated 1.9 (1.4; 3.0) cycles. The estimated maximal change from baseline at Week 12 was −0.6 (95% CI, –0.8; –0.4) (Fig. 5D), with 90% of the asymptote reached after an estimated 1.5 (95% CI, 1.1; 2.6) cycles. Although some differences between the models were observed for the estimated change from baseline during the double-blind cycle in Weeks 4 and 12 (Fig. 5C and D), overall both models were judged to be appropriate for the assessments.

### Active function (MFS overall score) assessment by age group

The estimated maximal change from baseline MFS overall score at Week 4 of any treatment cycle was 0.5 (95% CI, 0.3; 0.7) in patients aged ≤55 years and 0.7 (95% CI, 0.4; 1.0) in patients aged >55 years, with 90% of the asymptote reached after an estimated 4.3 (95% CI, 3.0; 7.4) cycles and 3.1 (95% CI, 2.3; 4.8) cycles, respectively (Supplementary Fig. 1A and B). At Week 12, the estimated maximal change from baseline was 0.6 (95% CI, 0.3; 1.0) in patients aged ≤55 years and 0.9 (95% CI, 0.6; 1.2) in patients aged >55 years, with 90% of the asymptote reached after an estimated 3.5 (95% CI, 2.4; 6.8) cycles and 3.6 (95% CI, 2.6; 5.8) cycles, respectively (Supplementary Fig. 1C and D).

## Discussion

The exploratory analyses presented here revealed incremental improvements with repeated injection cycles of abobotulinumtoxinA for upper limb spastic paresis, and the magnitude of improvements that could be expected before treatment effects plateau. These analyses also demonstrate the differing patterns of effect among efficacy parameters after injection: after repeated treatment cycles with abobotulinumtoxinA, the peak and plateau of the improvement of passive/perceived measures at Week 4 of a treatment cycle is around two to three injection cycles versus around four injection cycles for active measures. Also, in contrast to all other parameters, active function showed greater improvement at Week 12 compared with Week 4 after each injection cycle. These findings may provide physicians with valuable insights to inform treatment strategies for patients.

When the change from baseline to Week 4 of a treatment cycle was considered, around two to three cycles were indeed required to reach a near-maximal response to treatment for passive/perceived efficacy parameters, specifically: a mean 2.3 cycles for MAS in PTMG, 2.8 cycles for DAS PTT, 1.9 cycles for DAS limb position and 2.8 cycles for *X*_V3_ in extrinsic finger flexors. The near-maximal responses for these measures exceed those improvements observed at Week 4 after a single cycle during the double-blind study by about 50% (MAS in PTMG: −1.8 versus −1.2 and −1.4 for 500 and 1000 U; DAS PTT: −1.2 versus −0.7 for both 500 and 1000 U; *X*_V3_ in extrinsic finger flexors: 63.6° versus 39.3 and 47.7° for 500 and 1000 U, respectively) (Gracies *et al.*, 2015). The near-maximal response for DAS limb position at Week 4 (−0.7) was comparable to the previously reported changes across multiple injection cycles (−0.6 to −0.8 at Week 6, across four cycles) (Gordon *et al.*, 2004).

When compared with the passive/perceived efficacy parameters, a greater number of cycles were required to achieve a near-maximal treatment response at Week 4 of a treatment cycle for *X*_A_ in extrinsic finger flexors (mean, 4.2 cycles) and the MFS overall score (mean, 3.5 cycles). Although more cycles were required, these near-maximal improvements were almost twice those observed at Week 4 of the double-blind study (*X*_A_ in extrinsic finger flexors: 44.6 versus 23.9° and 17.6° for 500 and 1000 U; MFS overall: 0.6 versus 0.3 and 0.1 for 500 and 1000 U, respectively) (Gracies *et al.*, 2015). In a comparator study of a botulinum toxin type A (BoNT-A), tizanidine and placebo in upper limb spastic paresis, the change in MFS overall score after BoNT-A injection was numerically higher but not significantly different compared with the other groups at Week 6 after a single-treatment cycle (Gracies *et al.*, 2009). In the context of this study, this phenomenon may support the need for multiple injection cycles to reach maximal efficacy.

At Week 12 of a treatment cycle, around three to six cycles were necessary to reach a near-maximal response to treatment for most passive/perceived parameters, specifically: a mean of 6.4 cycles for MAS in PTMG, 3.2 cycles for DAS PTT and 3.6 cycles for *X*_V3_ in extrinsic finger flexors. The near-maximal response to treatment in terms of DAS limb position was achieved more rapidly, after a mean of 1.5 cycles. For active parameters, 3.7 cycles were required to achieve an estimated near maximal treatment response for MFS overall score, and 6.1 cycles for *X*_A_ in extrinsic finger flexors.

In many of the technical parameters assessed (*X*_A_ against the resistance of extrinsic finger flexors; *X*_V3_ [Tardieu scale] in extrinsic finger flexors and wrist flexors; *X*_V1_ in wrist flexors), a similar number of cycles were required to achieve a near-maximal treatment response at Weeks 4 and 12 of a treatment cycle; however, the magnitude of the responses was not as great at Week 12 versus 4, suggesting that for some active and passive parameters, a maximal technical effect of the treatment within a cycle may be achieved relatively quickly after injection, with the effect waning before the next cycle.

For the assessment of active function, the near-maximal treatment responses for MFS overall score at Week 12 of a treatment cycle were higher than the corresponding response at Week 4 (0.8 versus 0.6), suggesting that repeat abobotulinumtoxinA treatment not only maintained, but improved active function across the 12 weeks of each cycle. Functional improvements associated with repeat abobotulinumtoxinA injection over 12 weeks may represent a period of adjustment by the brain by which in the weeks after an injection, the brain ‘relearns’ functional tasks when muscles in the periphery are weakened or softened via abobotulinumtoxinA injection, to improve motor control. Therefore, 4 weeks may be inadequate for the brain to re-learn functional tasks completely, indicating that assessments made 4 weeks into a cycle will not have allowed sufficient time to capture the full effects of abobotulinumtoxinA on functional efficacy parameters in a clinical trial setting. A similar trend was observed in a meta-analysis, exploring the relationship between muscle tone and subjective functional benefits after a single abobotulinumtoxinA injection (Francis *et al.*, 2004). In the previously published study, the maximum change in tone preceded a maximal change in subjective arm function in one-third of the patients, in whom changes in both measures were observed, and it was suggested that patients would benefit from concurrent active rehabilitation to relearn use of muscles after tone reduction with abobotulinumtoxinA (Francis *et al.*, 2004).

The near-maximal treatment effect for the MFS overall score was also achieved after a similar number of cycles at Weeks 4 and 12 of a treatment cycle (a mean of 3.5 and 3.7 cycles, respectively), providing insight into the number of cycles that are likely to be required before the response to abobotulinumtoxinA may be expected to plateau for these parameters.

The magnitude of the near-maximal effect on active function after three to four repeated cycles of botulinum toxin injections is also important to take into consideration. As measured by the MFS, this maximum effect was between 0.6 and 0.8, i.e. above the minimal clinically important difference of 0.5 (Baude *et al.*, 2016), but this was not the case after only a single injection (Gracies *et al.*, 2015). This observation thus allows us to reflect on the design of prior placebo-controlled botulinum toxin studies that have explored the effects of single injections only. This might also explain the reason why it has been so difficult to show functional improvement with botulinum toxin injections over the years which, in turn, might encourage researchers in the future to design placebo-controlled studies that are better suited for demonstrating efficacy of botulinum toxin on active function (Sheean, 2001; Francis *et al.*, 2004), e.g., maintaining a placebo group across at least two injection cycles. Naturally, any study design containing a placebo control arm needs careful consideration, but in this context, if active function was viewed as the most important goal for a specific group of patients, it might then be considered ethically acceptable to maintain a placebo group beyond a single-treatment cycle.

When active function was considered by age group, patients aged >55 years had a slightly higher expected maximal effect compared with those aged ≤55 years, although the number of cycles required to reach this maximal effect was similar between age groups (3.6 cycles compared with 3.5 cycles, respectively). However, as a result of the overlap in 95% CIs, these differences between age groups should be interpreted with caution. The difference in expected maximal effect may be explained by the baseline scores for efficacy parameters, which suggested that on average, patients in the older age group were less severely affected in terms of active function and *X*_A_. As demonstrated by Pila *et al.* (2018), patients with hemiparesis who have more severe symptoms at baseline may be expected to show less progress over the long term (1 year here, compared with 4 years as described by Pila *et al.*, 2018); this highlights the importance of early intervention in the chronic phase after a stroke or traumatic brain injury. It is worth noting that baseline data from the present analyses also suggest that *X*_A_ is more strongly correlated with active function measures, compared with the MAS and DAS. This correlation between *X*_A_ and active function has also been demonstrated in a recent analysis of a new composite measure of *X*_A_ (Bayle *et al.*, 2020).

## Limitations

These exploratory analyses had several potential weaknesses, including: missing data as a source for possible bias; the absence of a placebo group and masking of treatment effect through use of concomitant therapy (as per standard of care, e.g. physiotherapy; oral anti-spastic medications). However, as discussed in the primary publications (Gracies *et al.*, 2015, 2018), potential sources of bias over time for individuals were minimized: community physiotherapy-initiated pre-study was permitted but had to remain unchanged during the study, where possible and any concomitant medications had to remain at the same dose throughout the trial. Furthermore, although the protocol required concomitant physiotherapy to remain consistent throughout these studies, the frequency and intensity of, or adherence to, therapy were not monitored. These analyses are based on partially set overall doses and the results might have differed in a real-life situation where the dose per muscle and overall dose injected are adjusted and vary. Finally, the CIs reported here were often large, suggesting great inter-individual variability in response.

## Conclusion

These exploratory analyses showed that the two statistical models were generally aligned and hence NLRC was suitable for assessing the estimated maximal efficacy and treatment duration required to reach it for most parameters. Unlike technical assessments, greater benefits in active function assessment outcomes were reached at Week 12 compared with Week 4 of a treatment cycle, suggesting that functional benefit to patients may have been underestimated in the previous studies using primary outcome measures at Week 4 post-injection. In addition, beneficial treatment responses for passive/perceived parameters reached their maximum between open-label Cycles 2 and 3, around one to two cycles before maximal responses in the active parameters investigated. The results of these analyses, particularly the clinically meaningful effects on active function beyond three injection cycles, suggest that treatment benefits may have been underestimated in the studies that terminated after one or two cycles (i.e. in much of the prior literature). Repeated treatment cycles with abobotulinumtoxinA in this chronically affected population can allow patients to relearn how to use the paretic arm, and could potentially be explored further in placebo-controlled trials that investigate active parameters as the primary outcome measures after at least three consecutive injection cycles.

## Supplementary material

Supplementary material is available at *Brain Communications* online.

## Supplementary Material

fcaa201_Supplementary_Data

## Abbreviations

BoNT-A =: botulinum toxin type A
CI =: confidence interval
DAS =: Disability Assessment Score
DB =: double blind
ITT =: intent to treat
MAS =: modified Ashworth scale
MFS =: Modified Frenchay Scale
MMRM: mixed-model repeated measures
NLRC =: non-linear random coefficient
OL =: open label
PTMG =: primary target muscle group
PTT =: principal target of treatment
*X*_A_ =: active range of motion
*X*_V1_ =: angle of arrest at slow speed (passive range of motion)
*X*_V3_ =: angle of catch at fast speed
