# Supplementary material for: When can maximal efficacy occur with repeat botulinum toxin injection in upper limb spastic paresis?
Source: Brain Commun. 2020 Nov 18;3(1):fcaa201. doi: 10.1093/braincomms/fcaa201 (PMC7850141; doi:10.1093/braincomms/fcaa201)
Supplement: fcaa201_Supplementary_Data [file fcaa201_supplementary_data.pdf]

Supplementary materials for:

When can maximal efficacy occur with repeat botulinum toxin injection in upper limb spastic paresis?

Jean-Michel Gracies, Robert Jech, Peter Valkovic, Philippe Marque, Michele Vecchio, Zoltan Denes, Claire Vilain, Bruno Delafont, Philippe Picaut

Supplementary Table 1. Baseline characteristics by age group (≤55 years or >55 years)

Characteristic	Patients aged ≤ 55 years	Patients aged > 55 years
Cause of spasticity, n	81	71
Stroke, n (%)	70 (86.4)	68 (95.8)
Traumatic brain injury, n (%)	11 (13.6)	3 (4.2)
MFS score, n	81	71
Mean (SD)	3.7 (1.3)	3.9 (1.5)
Median (min, max)	3.6 (1.3, 8.0)	3.8 (1.2, 7.6)
DAS score, n	80	71
Mean (SD)	2.5 (0.5)	2.6 (0.5)
Median (min, max)	3.0 (2.0, 3.0)	3.0 (2.0, 3.0)
MAS score, n	81	71
Mean (SD)	3.9 (0.4)	3.9 (0.4)
Median (min, max)	4.0 (3.0, 5.0)	4.0 (3.0, 5.0)
X_A – extrinsic finger flexors, n	75	63
Mean (SD)	52.3 (59.7)	68.7 (65.4)
Median (min, max)	20.0 (0.0, 210.0)	60.0 (0.0, 210.0)
X_A – wrist flexors, n	72	57
Mean (SD)	81.1 (39.1)	86.9 (35.8)
Median (min, max)	85.0 (0, 170.0)	90.0 (0.0, 160.0)
X_A – elbow flexors, n	64	52
Mean (SD)	135.6 (38.4)	142.2 (29.6)
Median (min, max)	140.0 (30.0, 180.0)	150.0 (65.0, 180.0)

DAS = Disability Assessment Scale; MAS = Modified Ashworth Scale; MFS, Modified Frenchay Scale; SD = standard deviation; X_A = active range of motion.

Supplementary Figure 1. Estimated mean change from baseline Modified Frenchay Scale (MFS) overall score, by A) patients aged ≤55 years at Week 4 of treatment cycle, B) patients aged >55 years at Week 4 of treatment cycle, C) patients aged ≤55 years at Week 12 of treatment cycle, D) patients aged >55 years at Week 12 of treatment cycle

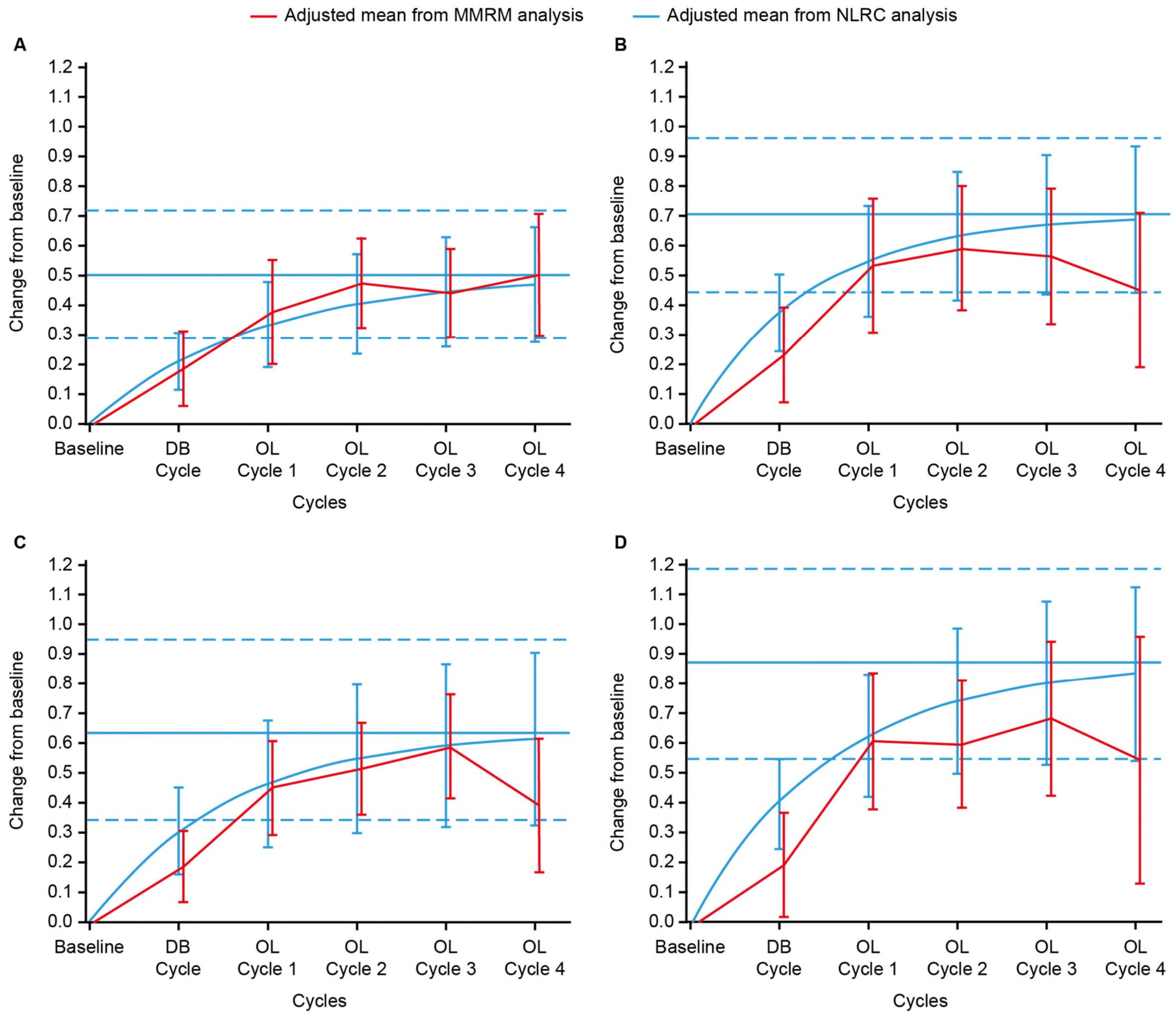

Displayed intervals are 95% CI. Horizontal blue lines correspond to the estimated asymptote from NLRC model, and its 95% CI.

CI = confidence interval; DB = double blind; MMRM = mixed model repeated measures; NLRC = non-linear random coefficient; OL = open label.
